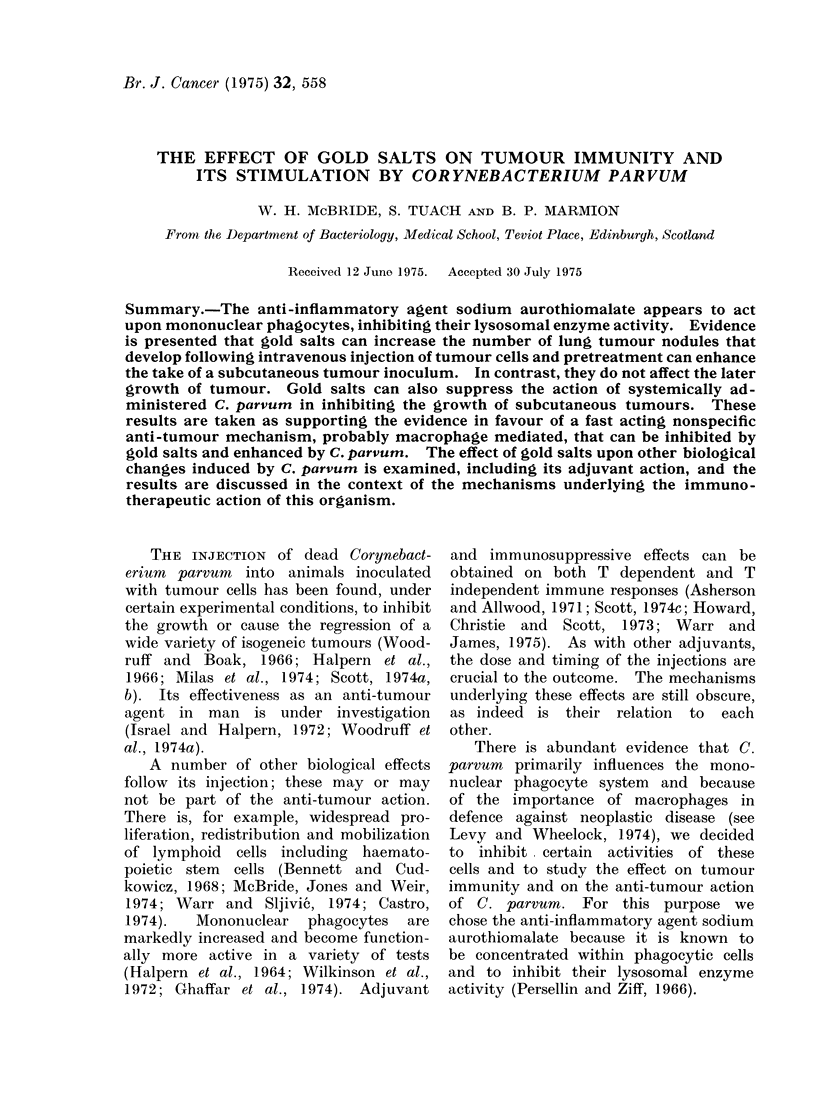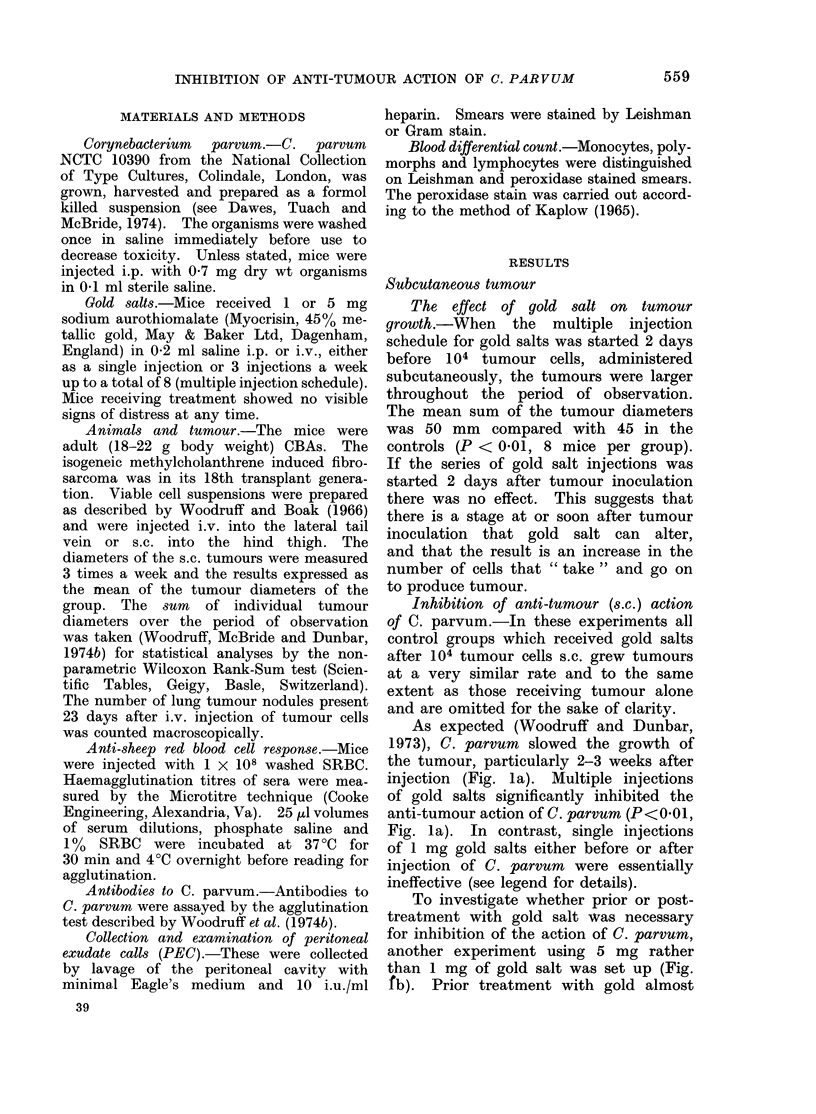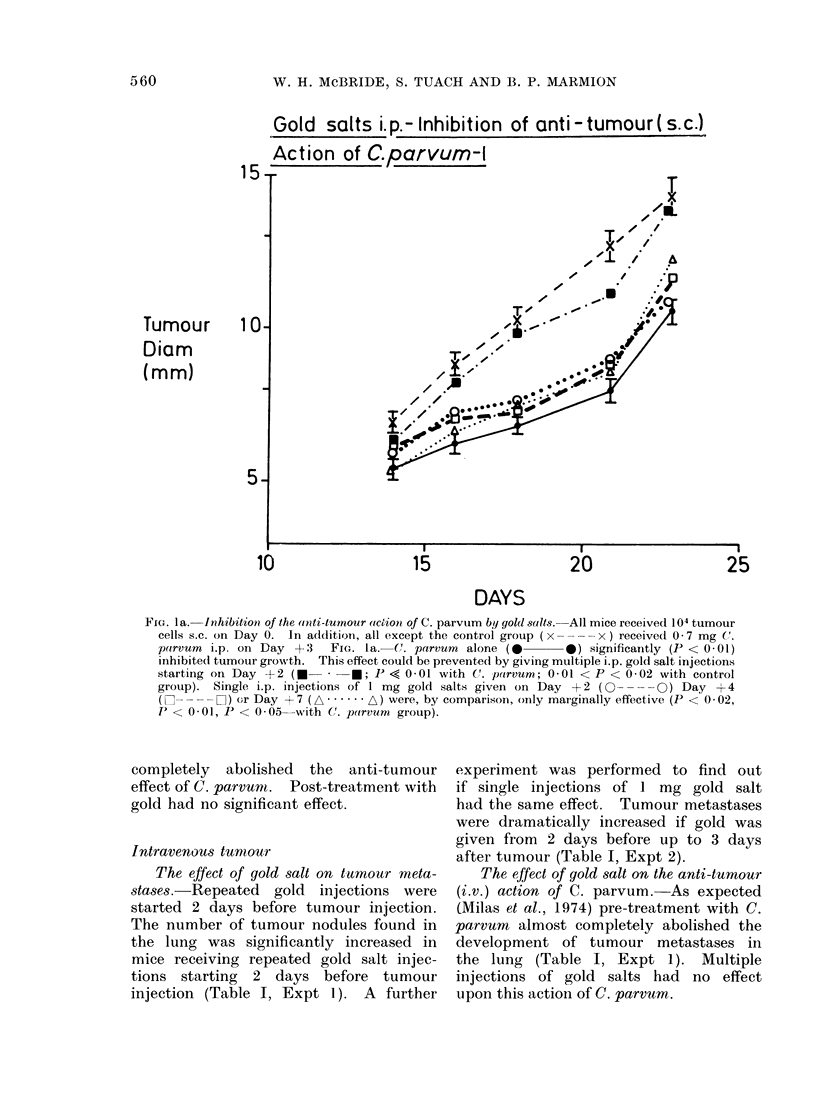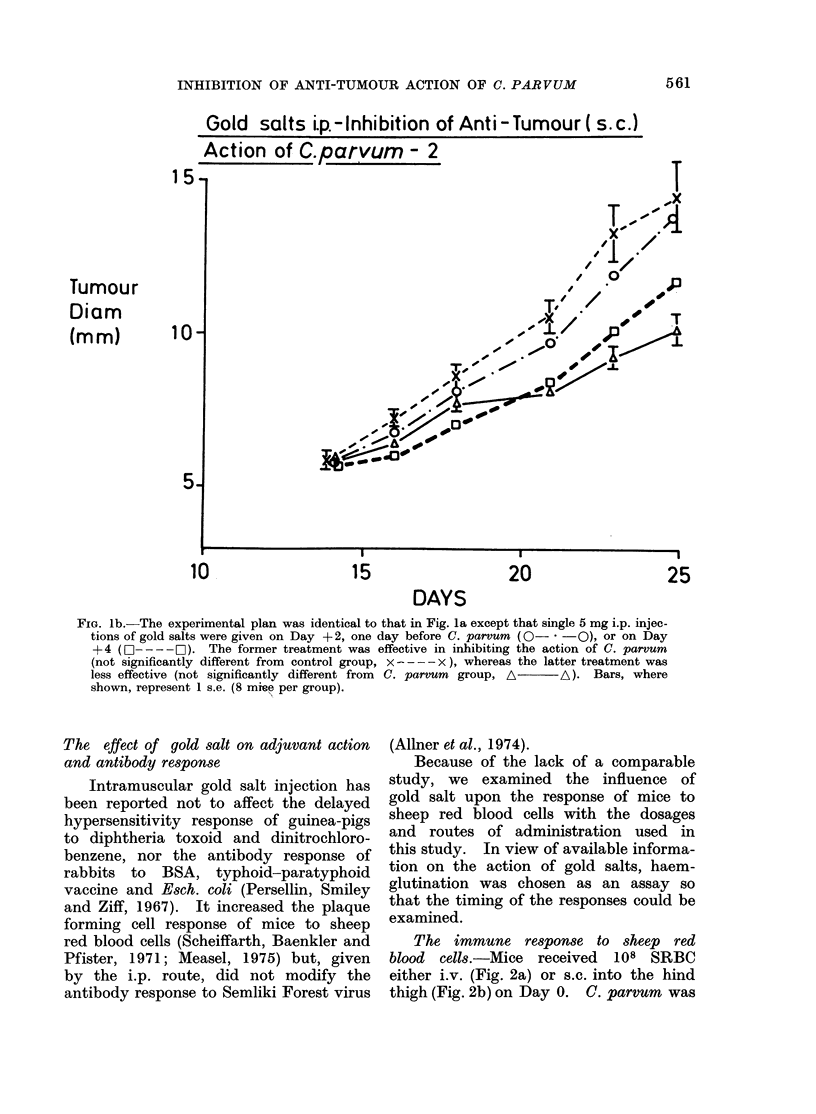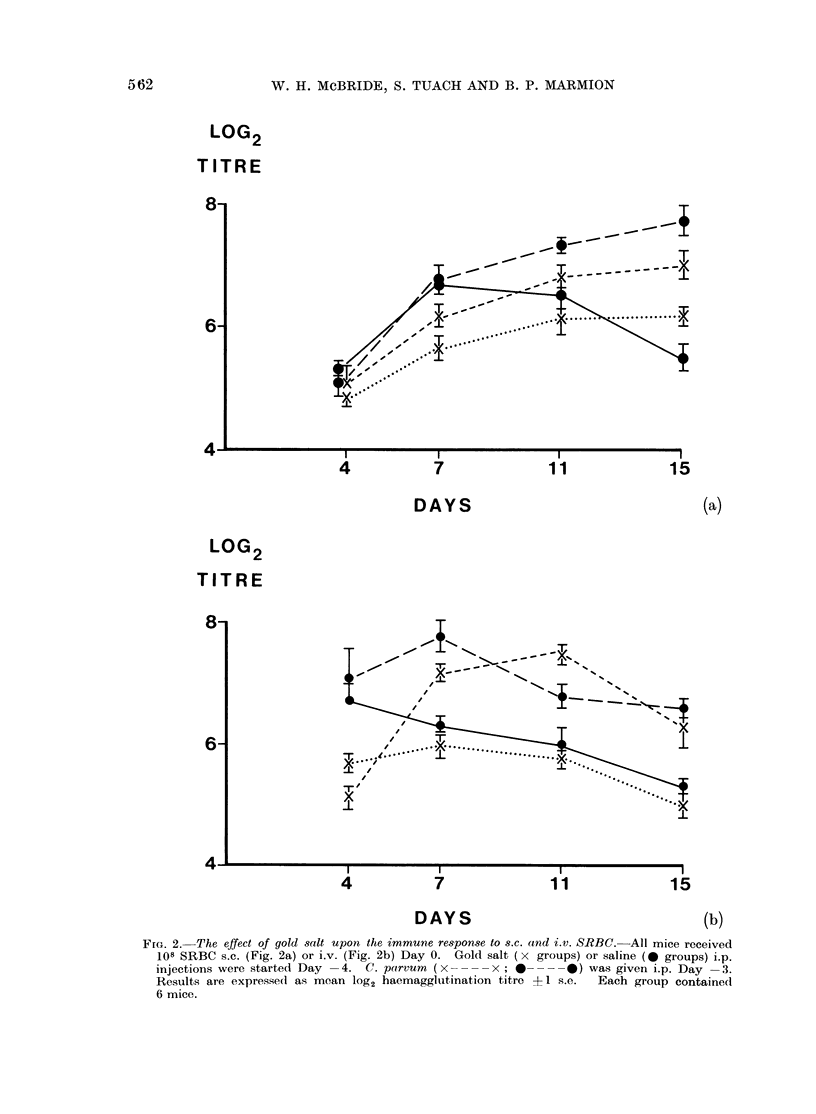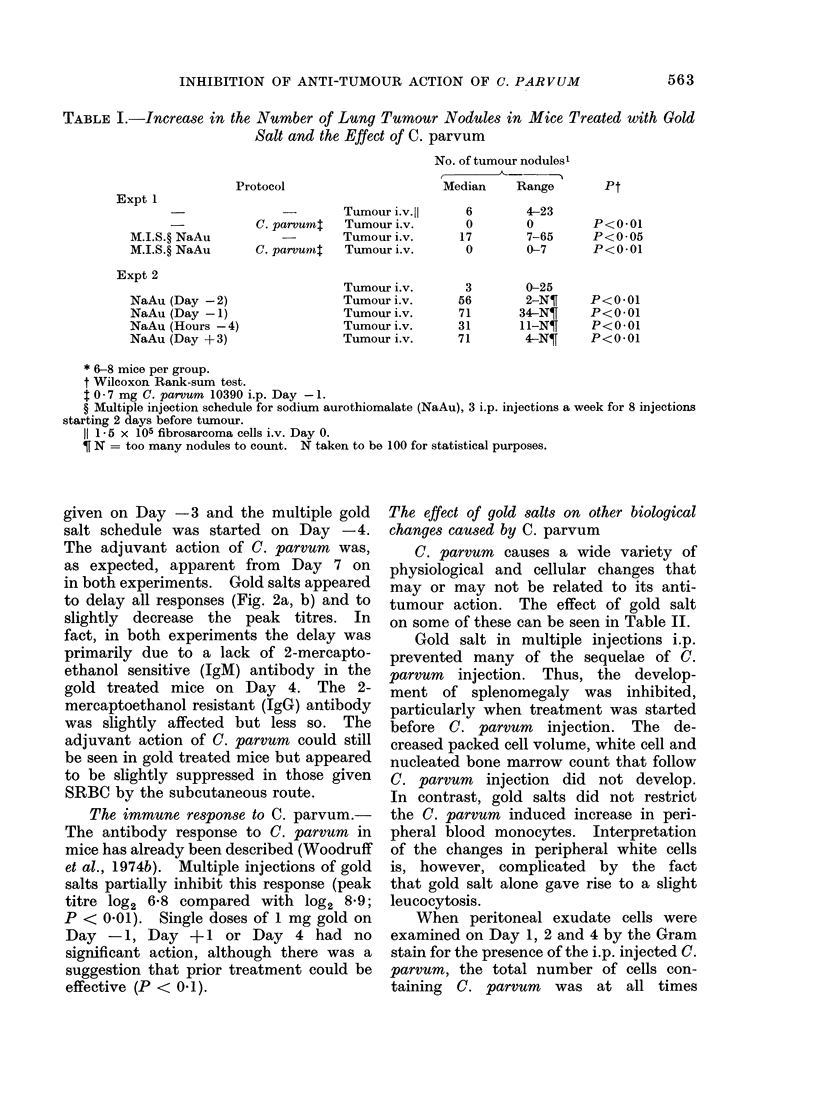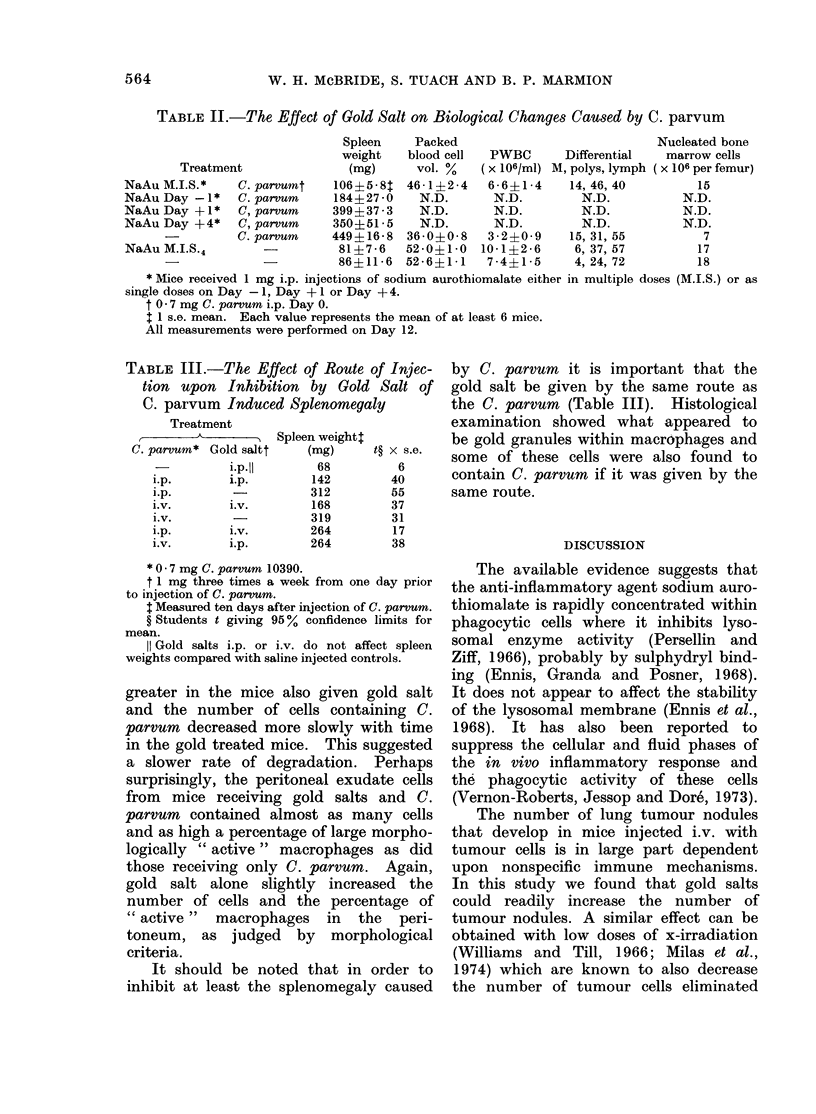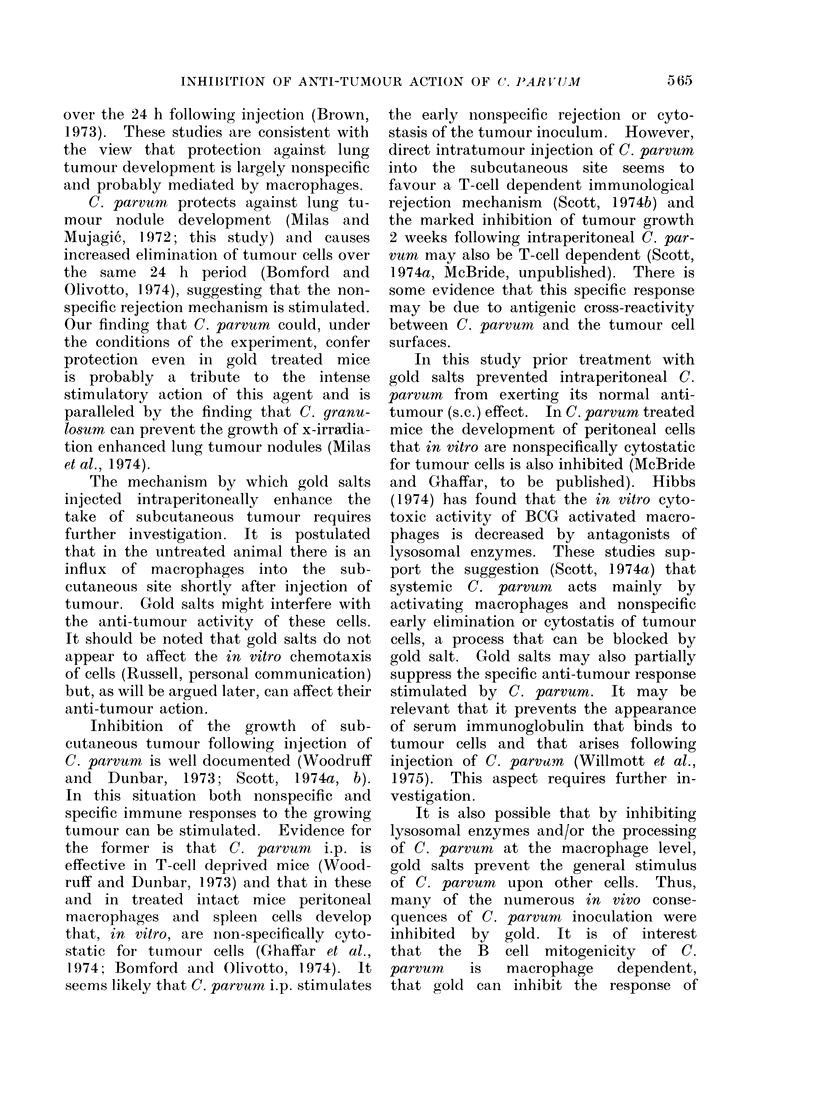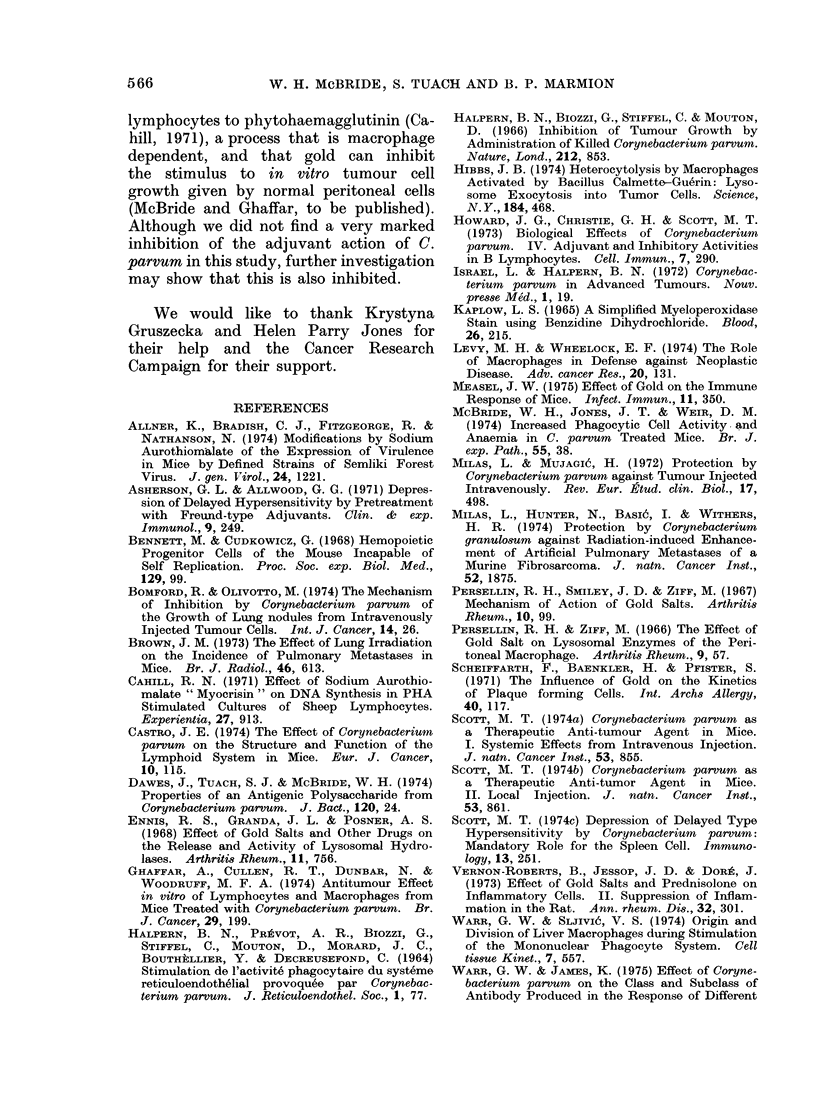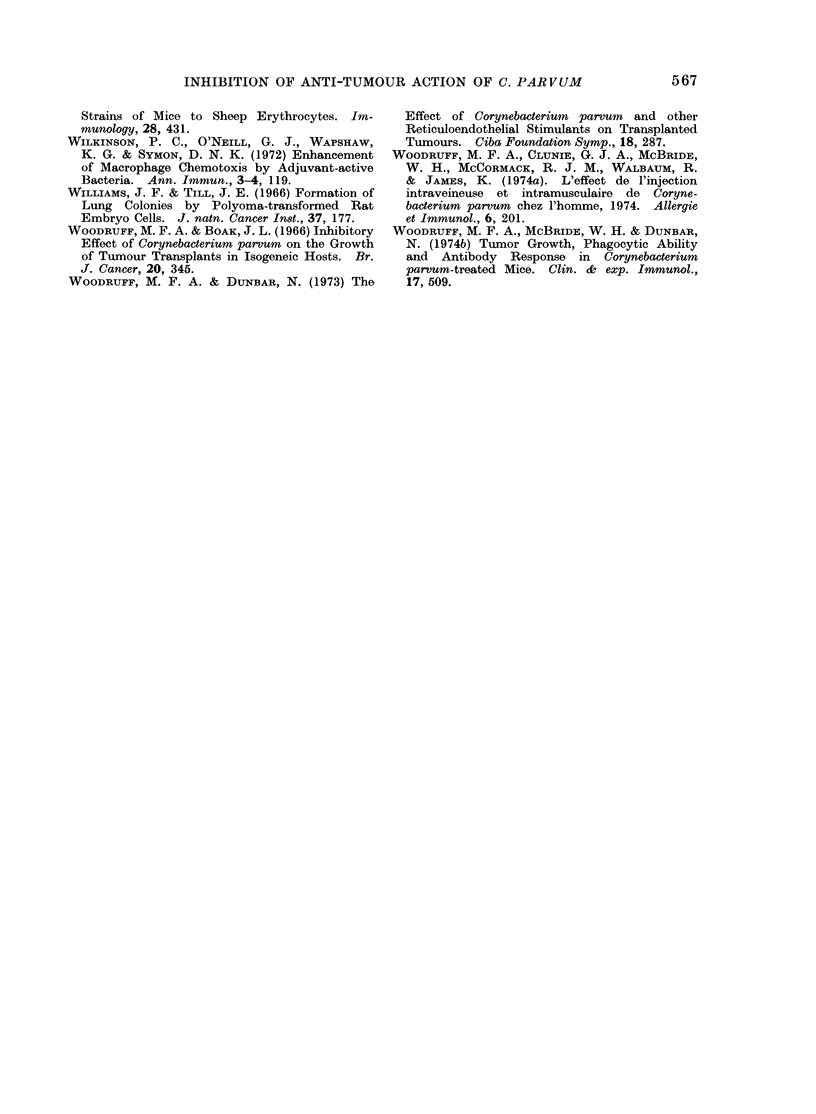# The effect of gold salts on tumour immunity and its stimulation by Corynebacterium Parvum.

**DOI:** 10.1038/bjc.1975.261

**Published:** 1975-11

**Authors:** W. H. McBride, S. Tuach, B. P. Marmion

## Abstract

The anti-inflammatory agent sodium aurothiomalate appears to act upon mononuclear phagocytes, inhibiting their lysosomal enzyme activity. Evidence is presented that gold salts can increase the number of lung tumour nodules that develop following intravenous injection of tumour cells and pretreatment can enhance the take of a subcutaneous tumour inoculum. In contrast, they do not affect the later growth of tumour. Gold salts can also suppress the action of systemically administered C. parvum in inhibiting the growth of subcutaneous tumours. These results are taken as supporting the evidence in favour of a fast acting nonspecific anti-tumour mechanism, probably macrophage mediated, that can be inhibited by gold salts and enhanced by C. parvum. The effect of gold salts upon other biological changes induced by C. parvum is examined, including its adjuvant action, and the results are discussed in the context of the mechanisms underlying the immunotherapeutic action of this organism.


*Br. J. Cancer* (1975) **32**, 558

### THE EFFECT OF GOLD SALTS ON TUMOUR IMMUNITY AND ITS STIMULATION BY *CORYNEBACTERIUM PARVUM*

W. H. McBRIDE, S. TUACH AND B. P. MARMION

*From the Department of Bacteriology, Medical School, Teviot Place, Edinburgh, Scotland*

Received 12 June 1975. Accepted 30 July 1975

**Summary.—The anti-inflammatory agent sodium aurothiomalate appears to act upon mononuclear phagocytes, inhibiting their lysosomal enzyme activity. Evidence is presented that gold salts can increase the number of lung tumour nodules that develop following intravenous injection of tumour cells and pretreatment can enhance the take of a subcutaneous tumour inoculum. In contrast, they do not affect the later growth of tumour. Gold salts can also suppress the action of systemically administered *C. parvum* in inhibiting the growth of subcutaneous tumours. These results are taken as supporting the evidence in favour of a fast acting nonspecific anti-tumour mechanism, probably macrophage mediated, that can be inhibited by gold salts and enhanced by *C. parvum*. The effect of gold salts upon other biological changes induced by *C. parvum* is examined, including its adjuvant action, and the results are discussed in the context of the mechanisms underlying the immunotherapeutic action of this organism.**

THE INJECTION of dead *Corynebacterium parvum* into animals inoculated with tumour cells has been found, under certain experimental conditions, to inhibit the growth or cause the regression of a wide variety of isogeneic tumours (Woodruff and Boak, 1966; Halpern *et al.*, 1966; Milas *et al.*, 1974; Scott, 1974*a*, *b*). Its effectiveness as an anti-tumour agent in man is under investigation (Israel and Halpern, 1972; Woodruff *et al.*, 1974*a*).

A number of other biological effects follow its injection; these may or may not be part of the anti-tumour action. There is, for example, widespread proliferation, redistribution and mobilization of lymphoid cells including haematopoietic stem cells (Bennett and Cudkowicz, 1968; McBride, Jones and Weir, 1974; Warr and Sljivić, 1974; Castro, 1974). Mononuclear phagocytes are markedly increased and become functionally more active in a variety of tests (Halpern *et al.*, 1964; Wilkinson *et al.*, 1972; Ghaffar *et al.*, 1974). Adjuvant and immunosuppressive effects can be obtained on both T dependent and T independent immune responses (Asherson and Allwood, 1971; Scott, 1974*c*; Howard, Christie and Scott, 1973; Warr and James, 1975). As with other adjuvants, the dose and timing of the injections are crucial to the outcome. The mechanisms underlying these effects are still obscure, as indeed is their relation to each other.

There is abundant evidence that *C. parvum* primarily influences the mononuclear phagocyte system and because of the importance of macrophages in defence against neoplastic disease (see Levy and Wheelock, 1974), we decided to inhibit certain activities of these cells and to study the effect on tumour immunity and on the anti-tumour action of *C. parvum*. For this purpose we chose the anti-inflammatory agent sodium aurothiomalate because it is known to be concentrated within phagocytic cells and to inhibit their lysosomal enzyme activity (Persellin and Ziff, 1966).

### MATERIALS AND METHODS

*Corynebacterium parvum.*—*C. parvum* NCTC 10390 from the National Collection of Type Cultures, Colindale, London, was grown, harvested and prepared as a formol killed suspension (see Dawes, Tuach and McBride, 1974). The organisms were washed once in saline immediately before use to decrease toxicity. Unless stated, mice were injected i.p. with 0·7 mg dry wt organisms in 0·1 ml sterile saline.

*Gold salts.*—Mice received 1 or 5 mg sodium aurothiomalate (Myocrisin, 45% metallic gold, May & Baker Ltd, Dagenham, England) in 0·2 ml saline i.p. or i.v., either as a single injection or 3 injections a week up to a total of 8 (multiple injection schedule). Mice receiving treatment showed no visible signs of distress at any time.

*Animals and tumour.*—The mice were adult (18–22 g body weight) CBAs. The isogeneic methylcholanthrene induced fibrosarcoma was in its 18th transplant generation. Viable cell suspensions were prepared as described by Woodruff and Boak (1966) and were injected i.v. into the lateral tail vein or s.c. into the hind thigh. The diameters of the s.c. tumours were measured 3 times a week and the results expressed as the mean of the tumour diameters of the group. The *sum* of individual tumour diameters over the period of observation was taken (Woodruff, McBride and Dunbar, 1974*b*) for statistical analyses by the non-parametric Wilcoxon Rank-Sum test (Scientific Tables, Geigy, Basle, Switzerland). The number of lung tumour nodules present 23 days after i.v. injection of tumour cells was counted macroscopically.

*Anti-sheep red blood cell response.*—Mice were injected with $1 \times 10^8$ washed SRBC. Haemagglutination titres of sera were measured by the Microtitre technique (Cooke Engineering, Alexandria, Va). 25 $\mu$l volumes of serum dilutions, phosphate saline and 1% SRBC were incubated at 37°C for 30 min and 4°C overnight before reading for agglutination.

*Antibodies to* C. parvum.—Antibodies to *C. parvum* were assayed by the agglutination test described by Woodruff *et al.* (1974*b*).

*Collection and examination of peritoneal exudate calls (PEC).*—These were collected by lavage of the peritoneal cavity with minimal Eagle's medium and 10 i.u./ml heparin. Smears were stained by Leishman or Gram stain.

*Blood differential count.*—Monocytes, polymorphs and lymphocytes were distinguished on Leishman and peroxidase stained smears. The peroxidase stain was carried out according to the method of Kaplow (1965).

### RESULTS

### *Subcutaneous tumour*

*The effect of gold salt on tumour growth.*—When the multiple injection schedule for gold salts was started 2 days before $10^4$ tumour cells, administered subcutaneously, the tumours were larger throughout the period of observation. The mean sum of the tumour diameters was 50 mm compared with 45 in the controls ($P < 0{\cdot}01$, 8 mice per group). If the series of gold salt injections was started 2 days after tumour inoculation there was no effect. This suggests that there is a stage at or soon after tumour inoculation that gold salt can alter, and that the result is an increase in the number of cells that " take " and go on to produce tumour.

*Inhibition of anti-tumour (s.c.) action of* C. parvum.—In these experiments all control groups which received gold salts after $10^4$ tumour cells s.c. grew tumours at a very similar rate and to the same extent as those receiving tumour alone and are omitted for the sake of clarity.

As expected (Woodruff and Dunbar, 1973), *C. parvum* slowed the growth of the tumour, particularly 2–3 weeks after injection (Fig. 1a). Multiple injections of gold salts significantly inhibited the anti-tumour action of *C. parvum* ($P<0{\cdot}01$, Fig. 1a). In contrast, single injections of 1 mg gold salts either before or after injection of *C. parvum* were essentially ineffective (see legend for details).

To investigate whether prior or post-treatment with gold salt was necessary for inhibition of the action of *C. parvum*, another experiment using 5 mg rather than 1 mg of gold salt was set up (Fig. 1b). Prior treatment with gold almost

FIG. 1a.—*Inhibition of the anti-tumour action of* C. parvum *by gold salts.*—All mice received $10^4$ tumour cells s.c. on Day 0. In addition, all except the control group (×– – – – ×) received 0·7 mg *C. parvum* i.p. on Day +3 FIG. 1a.—*C. parvum* alone (●———●) significantly ($P < 0{\cdot}01$) inhibited tumour growth. This effect could be prevented by giving multiple i.p. gold salt injections starting on Day +2 (■— · —■; $P \leqslant 0{\cdot}01$ with *C. parvum*; $0{\cdot}01 < P < 0{\cdot}02$ with control group). Single i.p. injections of 1 mg gold salts given on Day +2 (○– – – –○) Day +4 (□– – – –□) or Day +7 (△· · · · · ·△) were, by comparison, only marginally effective ($P < 0{\cdot}02$, $P < 0{\cdot}01$, $P < 0{\cdot}05$—with *C. parvum* group).

completely abolished the anti-tumour effect of *C. parvum*. Post-treatment with gold had no significant effect.

### *Intravenous tumour*

*The effect of gold salt on tumour metastases.*—Repeated gold injections were started 2 days before tumour injection. The number of tumour nodules found in the lung was significantly increased in mice receiving repeated gold salt injections starting 2 days before tumour injection (Table I, Expt 1). A further experiment was performed to find out if single injections of 1 mg gold salt had the same effect. Tumour metastases were dramatically increased if gold was given from 2 days before up to 3 days after tumour (Table I, Expt 2).

*The effect of gold salt on the anti-tumour (i.v.) action of* C. parvum.—As expected (Milas *et al.*, 1974) pre-treatment with *C. parvum* almost completely abolished the development of tumour metastases in the lung (Table I, Expt 1). Multiple injections of gold salts had no effect upon this action of *C. parvum*.

FIG. 1b.—The experimental plan was identical to that in Fig. 1a except that single 5 mg i.p. injections of gold salts were given on Day +2, one day before *C. parvum* (○— · —○), or on Day +4 (□- - - - -□). The former treatment was effective in inhibiting the action of *C. parvum* (not significantly different from control group, ×- - - - -×), whereas the latter treatment was less effective (not significantly different from *C. parvum* group, △———△). Bars, where shown, represent 1 s.e. (8 mice per group).

*The effect of gold salt on adjuvant action and antibody response*

Intramuscular gold salt injection has been reported not to affect the delayed hypersensitivity response of guinea-pigs to diphtheria toxoid and dinitrochlorobenzene, nor the antibody response of rabbits to BSA, typhoid–paratyphoid vaccine and *Esch. coli* (Persellin, Smiley and Ziff, 1967). It increased the plaque forming cell response of mice to sheep red blood cells (Scheiffarth, Baenkler and Pfister, 1971; Measel, 1975) but, given by the i.p. route, did not modify the antibody response to Semliki Forest virus (Allner *et al.*, 1974).

Because of the lack of a comparable study, we examined the influence of gold salt upon the response of mice to sheep red blood cells with the dosages and routes of administration used in this study. In view of available information on the action of gold salts, haemglutination was chosen as an assay so that the timing of the responses could be examined.

*The immune response to sheep red blood cells.*—Mice received $10^8$ SRBC either i.v. (Fig. 2a) or s.c. into the hind thigh (Fig. 2b) on Day 0. *C. parvum* was

FIG. 2.—*The effect of gold salt upon the immune response to s.c. and i.v. SRBC.*—All mice received $10^8$ SRBC s.c. (Fig. 2a) or i.v. (Fig. 2b) Day 0. Gold salt (× groups) or saline (● groups) i.p. injections were started Day −4. *C. parvum* (×– – – –×; ●– – – –●) was given i.p. Day −3. Results are expressed as mean $\log_2$ haemagglutination titre ±1 s.e. Each group contained 6 mice.

TABLE I.—*Increase in the Number of Lung Tumour Nodules in Mice Treated with Gold Salt and the Effect of* C. parvum

| Protocol | | | No. of tumour nodules[1] Median | Range | $P$† |
|---|---|---|---|---|---|
| Expt 1 | | | | | |
| — | — | Tumour i.v.‖ | 6 | 4–23 | |
| — | *C. parvum*‡ | Tumour i.v. | 0 | 0 | $P<0{\cdot}01$ |
| M.I.S.§ NaAu | — | Tumour i.v. | 17 | 7–65 | $P<0{\cdot}05$ |
| M.I.S.§ NaAu | *C. parvum*‡ | Tumour i.v. | 0 | 0–7 | $P<0{\cdot}01$ |
| Expt 2 | | | | | |
| | | Tumour i.v. | 3 | 0–25 | |
| NaAu (Day −2) | | Tumour i.v. | 56 | 2–N¶ | $P<0{\cdot}01$ |
| NaAu (Day −1) | | Tumour i.v. | 71 | 34–N¶ | $P<0{\cdot}01$ |
| NaAu (Hours −4) | | Tumour i.v. | 31 | 11–N¶ | $P<0{\cdot}01$ |
| NaAu (Day +3) | | Tumour i.v. | 71 | 4–N¶ | $P<0{\cdot}01$ |

* 6–8 mice per group.
† Wilcoxon Rank-sum test.
‡ 0·7 mg *C. parvum* 10390 i.p. Day −1.
§ Multiple injection schedule for sodium aurothiomalate (NaAu), 3 i.p. injections a week for 8 injections starting 2 days before tumour.
‖ $1{\cdot}5 \times 10^5$ fibrosarcoma cells i.v. Day 0.
¶ N = too many nodules to count. N taken to be 100 for statistical purposes.

given on Day −3 and the multiple gold salt schedule was started on Day −4. The adjuvant action of *C. parvum* was, as expected, apparent from Day 7 on in both experiments. Gold salts appeared to delay all responses (Fig. 2a, b) and to slightly decrease the peak titres. In fact, in both experiments the delay was primarily due to a lack of 2-mercapto-ethanol sensitive (IgM) antibody in the gold treated mice on Day 4. The 2-mercaptoethanol resistant (IgG) antibody was slightly affected but less so. The adjuvant action of *C. parvum* could still be seen in gold treated mice but appeared to be slightly suppressed in those given SRBC by the subcutaneous route.

*The immune response to* C. parvum.—The antibody response to *C. parvum* in mice has already been described (Woodruff *et al.*, 1974*b*). Multiple injections of gold salts partially inhibit this response (peak titre $\log_2$ 6·8 compared with $\log_2$ 8·9; $P < 0{\cdot}01$). Single doses of 1 mg gold on Day −1, Day +1 or Day 4 had no significant action, although there was a suggestion that prior treatment could be effective ($P < 0{\cdot}1$).

*The effect of gold salts on other biological changes caused by* C. parvum

*C. parvum* causes a wide variety of physiological and cellular changes that may or may not be related to its anti-tumour action. The effect of gold salt on some of these can be seen in Table II.

Gold salt in multiple injections i.p. prevented many of the sequelae of *C. parvum* injection. Thus, the development of splenomegaly was inhibited, particularly when treatment was started before *C. parvum* injection. The decreased packed cell volume, white cell and nucleated bone marrow count that follow *C. parvum* injection did not develop. In contrast, gold salts did not restrict the *C. parvum* induced increase in peripheral blood monocytes. Interpretation of the changes in peripheral white cells is, however, complicated by the fact that gold salt alone gave rise to a slight leucocytosis.

When peritoneal exudate cells were examined on Day 1, 2 and 4 by the Gram stain for the presence of the i.p. injected *C. parvum*, the total number of cells containing *C. parvum* was at all times

TABLE II.—*The Effect of Gold Salt on Biological Changes Caused by* C. parvum

| Treatment | | Spleen weight (mg) | Packed blood cell vol. % | PWBC ( × 10⁶/ml) | Differential M, polys, lymph | Nucleated bone marrow cells ( × 10⁶ per femur) |
|---|---|---|---|---|---|---|
| NaAu M.I.S.* | *C. parvum*† | 106±5·8‡ | 46·1±2·4 | 6·6±1·4 | 14, 46, 40 | 15 |
| NaAu Day −1* | *C. parvum* | 184±27·0 | N.D. | N.D. | N.D. | N.D. |
| NaAu Day +1* | *C, parvum* | 399±37·3 | N.D. | N.D. | N.D. | N.D. |
| NaAu Day +4* | *C, parvum* | 350±51·5 | N.D. | N.D. | N.D. | N.D. |
| — | *C. parvum* | 449±16·8 | 36·0±0·8 | 3·2±0·9 | 15, 31, 55 | 7 |
| NaAu M.I.S.$_4$ | — | 81±7·6 | 52·0±1·0 | 10·1±2·6 | 6, 37, 57 | 17 |
| — | — | 86±11·6 | 52·6±1·1 | 7·4±1·5 | 4, 24, 72 | 18 |

* Mice received 1 mg i.p. injections of sodium aurothiomalate either in multiple doses (M.I.S.) or as single doses on Day −1, Day +1 or Day +4.

† 0·7 mg *C. parvum* i.p. Day 0.

‡ 1 s.e. mean. Each value represents the mean of at least 6 mice.

All measurements were performed on Day 12.

TABLE III.—*The Effect of Route of Injection upon Inhibition by Gold Salt of* C. parvum *Induced Splenomegaly*

| Treatment: *C. parvum** | Treatment: Gold salt† | Spleen weight‡ (mg) | *t*§ × s.e. |
|---|---|---|---|
| — | i.p.‖ | 68 | 6 |
| i.p. | i.p. | 142 | 40 |
| i.p. | — | 312 | 55 |
| i.v. | i.v. | 168 | 37 |
| i.v. | — | 319 | 31 |
| i.p. | i.v. | 264 | 17 |
| i.v. | i.p. | 264 | 38 |

* 0·7 mg *C. parvum* 10390.

† 1 mg three times a week from one day prior to injection of *C. parvum.*

‡ Measured ten days after injection of *C. parvum.*

§ Students *t* giving 95% confidence limits for mean.

‖ Gold salts i.p. or i.v. do not affect spleen weights compared with saline injected controls.

greater in the mice also given gold salt and the number of cells containing *C. parvum* decreased more slowly with time in the gold treated mice. This suggested a slower rate of degradation. Perhaps surprisingly, the peritoneal exudate cells from mice receiving gold salts and *C. parvum* contained almost as many cells and as high a percentage of large morphologically " active " macrophages as did those receiving only *C. parvum.* Again, gold salt alone slightly increased the number of cells and the percentage of " active " macrophages in the peritoneum, as judged by morphological criteria.

It should be noted that in order to inhibit at least the splenomegaly caused by *C. parvum* it is important that the gold salt be given by the same route as the *C. parvum* (Table III). Histological examination showed what appeared to be gold granules within macrophages and some of these cells were also found to contain *C. parvum* if it was given by the same route.

### DISCUSSION

The available evidence suggests that the anti-inflammatory agent sodium aurothiomalate is rapidly concentrated within phagocytic cells where it inhibits lysosomal enzyme activity (Persellin and Ziff, 1966), probably by sulphydryl binding (Ennis, Granda and Posner, 1968). It does not appear to affect the stability of the lysosomal membrane (Ennis *et al.*, 1968). It has also been reported to suppress the cellular and fluid phases of the *in vivo* inflammatory response and the phagocytic activity of these cells (Vernon-Roberts, Jessop and Doré, 1973).

The number of lung tumour nodules that develop in mice injected i.v. with tumour cells is in large part dependent upon nonspecific immune mechanisms. In this study we found that gold salts could readily increase the number of tumour nodules. A similar effect can be obtained with low doses of x-irradiation (Williams and Till, 1966; Milas *et al.*, 1974) which are known to also decrease the number of tumour cells eliminated over the 24 h following injection (Brown, 1973). These studies are consistent with the view that protection against lung tumour development is largely nonspecific and probably mediated by macrophages.

*C. parvum* protects against lung tumour nodule development (Milas and Mujagić, 1972; this study) and causes increased elimination of tumour cells over the same 24 h period (Bomford and Olivotto, 1974), suggesting that the nonspecific rejection mechanism is stimulated. Our finding that *C. parvum* could, under the conditions of the experiment, confer protection even in gold treated mice is probably a tribute to the intense stimulatory action of this agent and is paralleled by the finding that *C. granulosum* can prevent the growth of x-irradiation enhanced lung tumour nodules (Milas *et al.*, 1974).

The mechanism by which gold salts injected intraperitoneally enhance the take of subcutaneous tumour requires further investigation. It is postulated that in the untreated animal there is an influx of macrophages into the subcutaneous site shortly after injection of tumour. Gold salts might interfere with the anti-tumour activity of these cells. It should be noted that gold salts do not appear to affect the *in vitro* chemotaxis of cells (Russell, personal communication) but, as will be argued later, can affect their anti-tumour action.

Inhibition of the growth of subcutaneous tumour following injection of *C. parvum* is well documented (Woodruff and Dunbar, 1973; Scott, 1974*a*, *b*). In this situation both nonspecific and specific immune responses to the growing tumour can be stimulated. Evidence for the former is that *C. parvum* i.p. is effective in T-cell deprived mice (Woodruff and Dunbar, 1973) and that in these and in treated intact mice peritoneal macrophages and spleen cells develop that, *in vitro*, are non-specifically cytostatic for tumour cells (Ghaffar *et al.*, 1974; Bomford and Olivotto, 1974). It seems likely that *C. parvum* i.p. stimulates the early nonspecific rejection or cytostasis of the tumour inoculum. However, direct intratumour injection of *C. parvum* into the subcutaneous site seems to favour a T-cell dependent immunological rejection mechanism (Scott, 1974*b*) and the marked inhibition of tumour growth 2 weeks following intraperitoneal *C. parvum* may also be T-cell dependent (Scott, 1974*a*, McBride, unpublished). There is some evidence that this specific response may be due to antigenic cross-reactivity between *C. parvum* and the tumour cell surfaces.

In this study prior treatment with gold salts prevented intraperitoneal *C. parvum* from exerting its normal anti-tumour (s.c.) effect. In *C. parvum* treated mice the development of peritoneal cells that *in vitro* are nonspecifically cytostatic for tumour cells is also inhibited (McBride and Ghaffar, to be published). Hibbs (1974) has found that the *in vitro* cytotoxic activity of BCG activated macrophages is decreased by antagonists of lysosomal enzymes. These studies support the suggestion (Scott, 1974*a*) that systemic *C. parvum* acts mainly by activating macrophages and nonspecific early elimination or cytostatis of tumour cells, a process that can be blocked by gold salt. Gold salts may also partially suppress the specific anti-tumour response stimulated by *C. parvum*. It may be relevant that it prevents the appearance of serum immunoglobulin that binds to tumour cells and that arises following injection of *C. parvum* (Willmott *et al.*, 1975). This aspect requires further investigation.

It is also possible that by inhibiting lysosomal enzymes and/or the processing of *C. parvum* at the macrophage level, gold salts prevent the general stimulus of *C. parvum* upon other cells. Thus, many of the numerous *in vivo* consequences of *C. parvum* inoculation were inhibited by gold. It is of interest that the B cell mitogenicity of *C. parvum* is macrophage dependent, that gold can inhibit the response of lymphocytes to phytohaemagglutinin (Cahill, 1971), a process that is macrophage dependent, and that gold can inhibit the stimulus to *in vitro* tumour cell growth given by normal peritoneal cells (McBride and Ghaffar, to be published). Although we did not find a very marked inhibition of the adjuvant action of *C. parvum* in this study, further investigation may show that this is also inhibited.

We would like to thank Krystyna Gruszecka and Helen Parry Jones for their help and the Cancer Research Campaign for their support.

### REFERENCES

ALLNER, K., BRADISH, C. J., FITZGEORGE, R. & NATHANSON, N. (1974) Modifications by Sodium Aurothiomalate of the Expression of Virulence in Mice by Defined Strains of Semliki Forest Virus. *J. gen. Virol.*, **24**, 1221.

ASHERSON, G. L. & ALLWOOD, G. G. (1971) Depression of Delayed Hypersensitivity by Pretreatment with Freund-type Adjuvants. *Clin. & exp. Immunol.*, **9**, 249.

BENNETT, M. & CUDKOWICZ, G. (1968) Hemopoietic Progenitor Cells of the Mouse Incapable of Self Replication. *Proc. Soc. exp. Biol. Med.*, **129**, 99.

BOMFORD, R. & OLIVOTTO, M. (1974) The Mechanism of Inhibition by *Corynebacterium parvum* of the Growth of Lung nodules from Intravenously Injected Tumour Cells. *Int. J. Cancer*, **14**, 26.

BROWN, J. M. (1973) The Effect of Lung Irradiation on the Incidence of Pulmonary Metastases in Mice. *Br. J. Radiol.*, **46**, 613.

CAHILL, R. N. (1971) Effect of Sodium Aurothiomalate "Myocrisin" on DNA Synthesis in PHA Stimulated Cultures of Sheep Lymphocytes. *Experientia*, **27**, 913.

CASTRO, J. E. (1974) The Effect of *Corynebacterium parvum* on the Structure and Function of the Lymphoid System in Mice. *Eur. J. Cancer*, **10**, 115.

DAWES, J., TUACH, S. J. & MCBRIDE, W. H. (1974) Properties of an Antigenic Polysaccharide from *Corynebacterium parvum*. *J. Bact.*, **120**, 24.

ENNIS, R. S., GRANDA, J. L. & POSNER, A. S. (1968) Effect of Gold Salts and Other Drugs on the Release and Activity of Lysosomal Hydrolases. *Arthritis Rheum.*, **11**, 756.

GHAFFAR, A., CULLEN, R. T., DUNBAR, N. & WOODRUFF, M. F. A. (1974) Antitumour Effect *in vitro* of Lymphocytes and Macrophages from Mice Treated with *Corynebacterium parvum*. *Br. J. Cancer*, **29**, 199.

HALPERN, B. N., PRÉVOT, A. R., BIOZZI, G., STIFFEL, C., MOUTON, D., MORARD, J. C., BOUTHÈLLIER, Y. & DECREUSEFOND, C. (1964) Stimulation de l'activité phagocytaire du systéme reticuloendothélial provoquée par *Corynebacterium parvum*. *J. Reticuloendothel. Soc.*, **1**, 77.

HALPERN, B. N., BIOZZI, G., STIFFEL, C. & MOUTON, D. (1966) Inhibition of Tumour Growth by Administration of Killed *Corynebacterium parvum*. *Nature, Lond.*, **212**, 853.

HIBBS, J. B. (1974) Heterocytolysis by Macrophages Activated by Bacillus Calmette–Guérin: Lysosome Exocytosis into Tumor Cells. *Science, N.Y.*, **184**, 468.

HOWARD, J. G., CHRISTIE, G. H. & SCOTT, M. T. (1973) Biological Effects of *Corynebacterium parvum*. IV. Adjuvant and Inhibitory Activities in B Lymphocytes. *Cell. Immun.*, **7**, 290.

ISRAEL, L. & HALPERN, B. N. (1972) *Corynebacterium parvum* in Advanced Tumours. *Nouv. presse Méd.*, **1**, 19.

KAPLOW, L. S. (1965) A Simplified Myeloperoxidase Stain using Benzidine Dihydrochloride. *Blood*, **26**, 215.

LEVY, M. H. & WHEELOCK, E. F. (1974) The Role of Macrophages in Defense against Neoplastic Disease. *Adv. cancer Res.*, **20**, 131.

MEASEL, J. W. (1975) Effect of Gold on the Immune Response of Mice. *Infect. Immun.*, **11**, 350.

MCBRIDE, W. H., JONES, J. T. & WEIR, D. M. (1974) Increased Phagocytic Cell Activity and Anaemia in *C. parvum* Treated Mice. *Br. J. exp. Path.*, **55**, 38.

MILAS, L. & MUJAGIĆ, H. (1972) Protection by *Corynebacterium parvum* against Tumour Injected Intravenously. *Rev. Eur. Étud. clin. Biol.*, **17**, 498.

MILAS, L., HUNTER, N., BASIĆ, I. & WITHERS, H. R. (1974) Protection by *Corynebacterium granulosum* against Radiation-induced Enhancement of Artificial Pulmonary Metastases of a Murine Fibrosarcoma. *J. natn. Cancer Inst.*, **52**, 1875.

PERSELLIN, R. H., SMILEY, J. D. & ZIFF, M. (1967) Mechanism of Action of Gold Salts. *Arthritis Rheum.*, **10**, 99.

PERSELLIN, R. H. & ZIFF, M. (1966) The Effect of Gold Salt on Lysosomal Enzymes of the Peritoneal Macrophage. *Arthritis Rheum.*, **9**, 57.

SCHEIFFARTH, F., BAENKLER, H. & PFISTER, S. (1971) The Influence of Gold on the Kinetics of Plaque forming Cells. *Int. Archs Allergy*, **40**, 117.

SCOTT, M. T. (1974*a*) *Corynebacterium parvum* as a Therapeutic Anti-tumour Agent in Mice. I. Systemic Effects from Intravenous Injection. *J. natn. Cancer Inst.*, **53**, 855.

SCOTT, M. T. (1974*b*) *Corynebacterium parvum* as a Therapeutic Anti-tumor Agent in Mice. II. Local Injection. *J. natn. Cancer Inst.*, **53**, 861.

SCOTT, M. T. (1974*c*) Depression of Delayed Type Hypersensitivity by *Corynebacterium parvum*: Mandatory Role for the Spleen Cell. *Immunology*, **13**, 251.

VERNON-ROBERTS, B., JESSOP, J. D. & DORÉ, J. (1973) Effect of Gold Salts and Prednisolone on Inflammatory Cells. II. Suppression of Inflammation in the Rat. *Ann. rheum. Dis.*, **32**, 301.

WARR, G. W. & SLJIVIĆ, V. S. (1974) Origin and Division of Liver Macrophages during Stimulation of the Mononuclear Phagocyte System. *Cell tissue Kinet.*, **7**, 557.

WARR, G. W. & JAMES, K. (1975) Effect of *Corynebacterium parvum* on the Class and Subclass of Antibody Produced in the Response of Different

Strains of Mice to Sheep Erythrocytes. *Immunology*, **28**, 431.

WILKINSON, P. C., O'NEILL, G. J., WAPSHAW, K. G. & SYMON, D. N. K. (1972) Enhancement of Macrophage Chemotoxis by Adjuvant-active Bacteria. *Ann. Immun.*, **3–4**, 119.

WILLIAMS, J. F. & TILL, J. E. (1966) Formation of Lung Colonies by Polyoma-transformed Rat Embryo Cells. *J. natn. Cancer Inst.*, **37**, 177.

WOODRUFF, M. F. A. & BOAK, J. L. (1966) Inhibitory Effect of *Corynebacterium parvum* on the Growth of Tumour Transplants in Isogeneic Hosts. *Br. J. Cancer*, **20**, 345.

WOODRUFF, M. F. A. & DUNBAR, N. (1973) The Effect of *Corynebacterium parvum* and other Reticuloendothelial Stimulants on Transplanted Tumours. *Ciba Foundation Symp.*, **18**, 287.

WOODRUFF, M. F. A., CLUNIE, G. J. A., MCBRIDE, W. H., MCCORMACK, R. J. M., WALBAUM, R. & JAMES, K. (1974*a*). L'effect de l'injection intraveineuse et intramusculaire de *Corynebacterium parvum* chez l'homme, 1974. *Allergie et Immunol.*, **6**, 201.

WOODRUFF, M. F. A., MCBRIDE, W. H. & DUNBAR, N. (1974*b*) Tumor Growth, Phagocytic Ability and Antibody Response in *Corynebacterium parvum*-treated Mice. *Clin. & exp. Immunol.*, **17**, 509.